# Di­chlorido­(*N*,*N*-diethyl-4-{[(quinolin-2-yl)methyl­idene]amino-κ^2^ *N*,*N*′}aniline)mercury(II)

**DOI:** 10.1107/S160053681400957X

**Published:** 2014-05-03

**Authors:** Md. Serajul Haque Faizi, Sahid Hussain

**Affiliations:** a Department of Chemistry, Indian Institute of Technology Kanpur, Kanpur, UP 208 016, India; b Department of Chemistry, Indian Institute of Technology Patna, Patna, Bihar 800 013, India

## Abstract

In the mononuclear title complex, [HgCl_2_(C_20_H_21_N_3_)], synthesized from the quinoline-derived Schiff base *N* ^1^,*N* ^1^-diethyl-*N* ^4^-(quinolin-2-yl­methyl­idene)benzene-1,4-di­amine (QMBD), the coordination geometry around the Hg^2+^ atom is distorted tetra­hedral, comprising two Cl atoms [Hg—Cl = 2.3654 (19) and 2.4394 (18) Å] and two N-atom donors from the QMBD ligand, *viz.* one imine and quinoline [Hg—N = 2.334 (5) and 2.340 (5) Å, respectively]. In the crystal, weak C—H⋯Cl hydrogen bonds and weak π–π aromatic ring stacking inter­actions [minimum ring-centroid separation = 3.680 (4) Å] give an overall three-dimensional network.

## Related literature   

For applications of quinolyl imine and related structures, see: Mandal *et al.* (2012 ▶); Motswainyana *et al.* (2013 ▶); Das *et al.* (2013 ▶); Song *et al.* (2011 ▶); Jursic *et al.* (2002 ▶); Marjani *et al.* (2009 ▶); Faizi & Sen (2014 ▶).
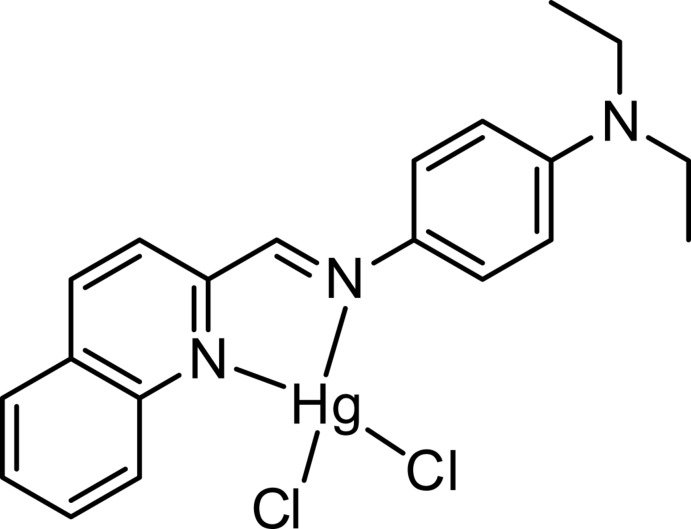


## Experimental   

### 

#### Crystal data  

[HgCl_2_(C_20_H_21_N_3_)]
*M* *_r_* = 574.89
Monoclinic, 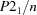

*a* = 8.8522 (19) Å
*b* = 9.474 (2) Å
*c* = 23.512 (5) Å
β = 97.446 (4)°
*V* = 1955.2 (7) Å^3^
*Z* = 4
Mo *K*α radiation
μ = 8.15 mm^−1^
*T* = 100 K
0.26 × 0.18 × 0.13 mm

#### Data collection  

Bruker SMART APEX CCD diffractometer
Absorption correction: multi-scan (*SADABS*; Sheldrick, 2004 ▶) *T*_min_ = 0.226, *T*_max_ = 0.417
9875 measured reflections
3438 independent reflections
2979 reflections with *I* > 2σ(*I*)
*R*_int_ = 0.034

#### Refinement  

*R*[*F* ^2^ > 2σ(*F* ^2^)] = 0.029
*wR*(*F* ^2^) = 0.089
*S* = 1.17
3438 reflections
233 parameters
H atoms treated by a mixture of independent and constrained refinement
Δρ_max_ = 1.91 e Å^−3^
Δρ_min_ = −1.07 e Å^−3^

### 

Data collection: *SMART* (Bruker, 2003 ▶); cell refinement: *SAINT* (Bruker, 2003 ▶); data reduction: *SAINT*; program(s) used to solve structure: *SIR97* (Altomare *et al.*, 1999 ▶); program(s) used to refine structure: *SHELXL97* (Sheldrick, 2008 ▶); molecular graphics: *DIAMOND* (Brandenburg & Putz, 2006 ▶); software used to prepare material for publication: *DIAMOND*.

## Supplementary Material

Crystal structure: contains datablock(s) global, I. DOI: 10.1107/S160053681400957X/zs2295sup1.cif

Structure factors: contains datablock(s) I. DOI: 10.1107/S160053681400957X/zs2295Isup2.hkl

CCDC reference: 999849

Additional supporting information:  crystallographic information; 3D view; checkCIF report

## Figures and Tables

**Table 1:** Hydrogen-bond geometry (Å, °)

*D*—H⋯*A*	*D*—H	H⋯*A*	*D*⋯*A*	*D*—H⋯*A*
C12—H12⋯Cl2^i^	0.93	2.82	3.703 (7)	159
C15—H15⋯Cl1^ii^	0.93	2.82	3.715 (7)	162

Symmetry codes: (i) 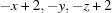; (ii) 
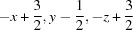
.

**Fig. 1.**
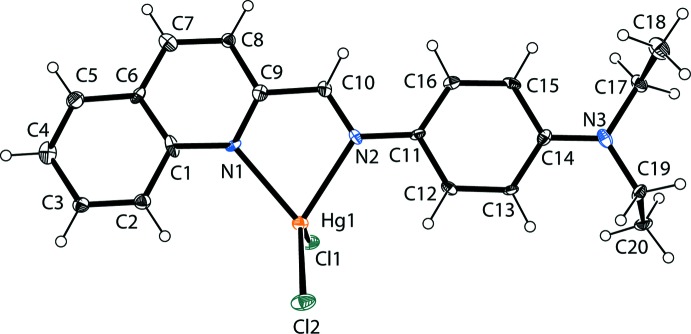
The molecular conformation and atom-numbering scheme for the title complex with non-H atoms drawn as 30% probability displacement ellipsoids.

**Fig. 2.**
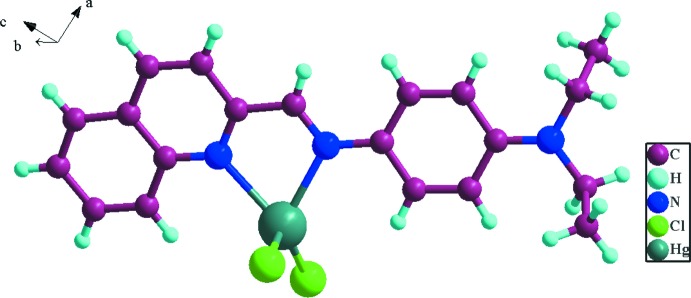
A further view of the title complex.

**Fig. 3.**
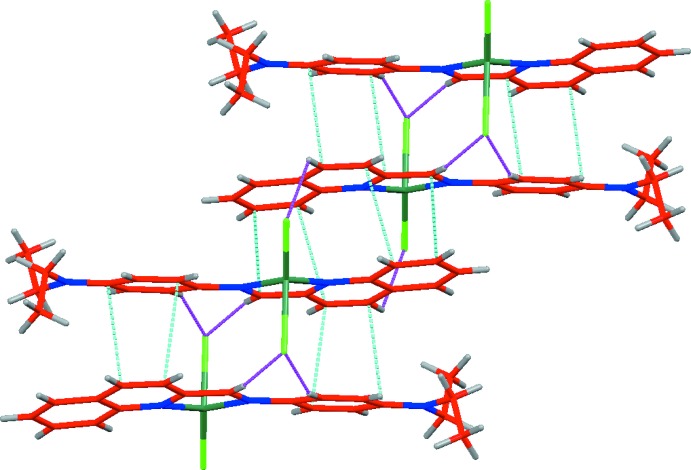
The one-dimensional weak interactive chain structure in the title complex extending along the approximate c-cell direction, with associations shown as dashed lines.

**Fig. 4.**
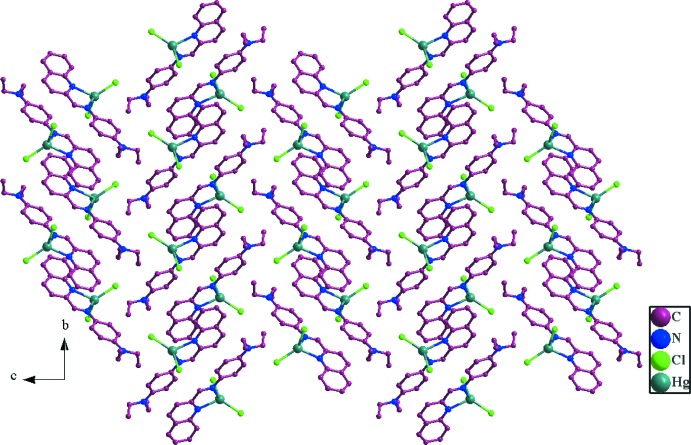
The structure viewed along the a-cell direction.

## supplementary crystallographic information

### 1. Comment

Mercury is one of the most prevalent toxic metals in the environment and gains access to the body orally or dermally, causing cell dysfunction that consequently leads to health problems (Mandal *et al.*, 2012). Quinolyl derivatives of Schiff bases are important building blocks for many important compounds widely used in biological applications such as antioxidative, anticancer, fluorescent probe agents in industry, in coordination chemistry and in catalysis (Motswainyana *et al.*, 2013; Das *et al.*, 2013; Song *et al.*, 2011; Jursic *et al.*, 2002). The synthesis of a complex of mercury(II) using the quinoline aldehyde derivative of the Schiff base *N*^1^,*N*^1^-diethyl-*N*^4^- (quinolin-2-ylmethylene-1,4-diamine (QMBD) has not previously been reported. The title Hg^II^ complex with QMBD, [Hg(C_20_H_21_N_3_)Cl_2_] has now been synthesized and the structure is reported herein.

In the title mononuclear complex (Figs. 1, 2) the HgCl_2_N_2_ coordination geometry is distorted tetrahedral, comprising two Cl-atoms [Hg1—Cl1 and Hg1—Cl2 = 2.3654 (19) and 2.4394 (18) Å respectively] and two N-atom donors from the QMBD ligand, one imine [Hg1–N2 = 2.334 (5) Å] and the other quinoline [Hg1—N1 = 2.340 (5) Å]. The observed Hg—Cl and Hg—N bond lengths and bond angles are considered normal for this type of Hg^II^ complex, *e.g.* (Marjani *et al.*, 2009; Faizi & Sen, 2014). In the crystal, weak C12—H···Cl2^i^ [3.703 (7) Å] and C15—H···Cl1^ii^ [3.715 (7) Å hydrogen bonds (Table 1) and π–π aromatic ring stacking interactions [minimum ring centroid separation between the quinoline ring moiety defined by atoms N1–C9 = 3.680 (4) Å] give an overall three-dimensional framework structure (Figs. 3, 4).

### 2. Experimental

The iminoquinolyl compound *N*^1^,*N*^1^-diethyl-*N*^4^-(quinolin-2-ylmethylidene)benzene-1,4-diamine (QMBD) was prepared by reacting 2-quinolinecarboxaldehyde with a substituted aniline and was obtained in very good yields. This compound was characterized by FT—IR, NMR and ESI-Mass spectroscopy. A mixture of QMBD (0.10 g, 0.33 mmol), mercury(II) chloride (0.09 g, 0.33 mmol) and ethanol (5 ml) were stirred vigorously for 1 h, after which the precipitate was filtered off and redissolved in dimethylformamide. Crystals of the title complex suitable for X-ray analysis was obtained within 3 days by slow evaporation of the DMF solvent.

### 3. Refinement

All H-atoms were positioned geometrically and refined using a riding model with C—H = 0.90–0.93 Å (aromatic), 0.97 Å (methylene) or 0.96 Å (methyl) and with *U*_iso_(H) = 1.2*U*_eq_(C) or 1.5*U*_eq_(C) (methyl). A large residual electron density peak (1.78 eÅ^-3^) located 1.47 from C19 of one of the ethyl goups suggested minor methyl group orientational disorder but this was not modelled.

### Crystal data

**Table:**

[HgCl_2_(C_20_H_21_N_3_)]	*F*(000) = 1104
*M**_r_* = 574.89	*D*_x_ = 1.953 Mg m^−^^3^
Monoclinic, *P*2_1_/*n*	Mo *K*α radiation, λ = 0.71073 Å
Hall symbol: -P 2yn	Cell parameters from 999 reflections
*a* = 8.8522 (19) Å	θ = 2.1–28.2°
*b* = 9.474 (2) Å	µ = 8.15 mm^−^^1^
*c* = 23.512 (5) Å	*T* = 100 K
β = 97.446 (4)°	Block, yellow
*V* = 1955.2 (7) Å^3^	0.26 × 0.18 × 0.13 mm
*Z* = 4	

### Data collection

**Table:**

Bruker SMART APEX CCD diffractometer	3438 independent reflections
Radiation source: fine-focus sealed tube	2979 reflections with *I* > 2σ(*I*)
Graphite monochromator	*R*_int_ = 0.034
ω scans	θ_max_ = 25.0°, θ_min_ = 1.8°
Absorption correction: multi-scan (*SADABS*; Sheldrick, 2004)	*h* = −10→7
*T*_min_ = 0.226, *T*_max_ = 0.417	*k* = −11→11
9875 measured reflections	*l* = −27→27

### Refinement

**Table:**

Refinement on *F*^2^	Primary atom site location: structure-invariant direct methods
Least-squares matrix: full	Secondary atom site location: difference Fourier map
*R*[*F*^2^ > 2σ(*F*^2^)] = 0.029	Hydrogen site location: inferred from neighbouring sites
*wR*(*F*^2^) = 0.089	H atoms treated by a mixture of independent and constrained refinement
*S* = 1.17	*w* = 1/[σ^2^(*F*_o_^2^) + (0.0308*P*)^2^ + 10.3789*P*] where *P* = (*F*_o_^2^ + 2*F*_c_^2^)/3
3438 reflections	(Δ/σ)_max_ < 0.001
233 parameters	Δρ_max_ = 1.91 e Å^−^^3^
0 restraints	Δρ_min_ = −1.07 e Å^−^^3^

### Special details

**Table:**

Geometry. All e.s.d.'s (except the e.s.d. in the dihedral angle between two l.s. planes) are estimated using the full covariance matrix. The cell e.s.d.'s are taken into account individually in the estimation of e.s.d.'s in distances, angles and torsion angles; correlations between e.s.d.'s in cell parameters are only used when they are defined by crystal symmetry. An approximate (isotropic) treatment of cell e.s.d.'s is used for estimating e.s.d.'s involving l.s. planes.
Refinement. Refinement of *F*^2^ against ALL reflections. The weighted *R*-factor *wR* and goodness of fit *S* are based on *F*^2^, conventional *R*-factors *R* are based on *F*, with *F* set to zero for negative *F*^2^. The threshold expression of *F*^2^ > σ(*F*^2^) is used only for calculating *R*-factors(gt) *etc*. and is not relevant to the choice of reflections for refinement. *R*-factors based on *F*^2^ are statistically about twice as large as those based on *F*, and *R*- factors based on ALL data will be even larger.

### Fractional atomic coordinates and isotropic or equivalent isotropic displacement parameters (Å^2^)

**Table:**

	*x*	*y*	*z*	*U*_iso_*/*U*_eq_	
C1	0.8207 (7)	0.4421 (7)	1.0240 (3)	0.0184 (10)	
C2	0.6971 (8)	0.5234 (7)	0.9970 (3)	0.0225 (15)	
H2	0.6564	0.5035	0.9594	0.027*	
C3	0.6374 (8)	0.6313 (7)	1.0260 (3)	0.0253 (16)	
H3	0.5575	0.6853	1.0078	0.030*	
C4	0.6962 (8)	0.6602 (7)	1.0826 (3)	0.0221 (15)	
H4	0.6540	0.7326	1.1021	0.027*	
C5	0.8150 (9)	0.5836 (7)	1.1100 (3)	0.0268 (16)	
H5	0.8521	0.6041	1.1479	0.032*	
C6	0.8819 (8)	0.4740 (7)	1.0813 (3)	0.0218 (15)	
C7	1.0050 (8)	0.3942 (6)	1.1069 (3)	0.0211 (14)	
H7	1.0451	0.4107	1.1448	0.025*	
C8	1.0667 (7)	0.2912 (7)	1.0759 (3)	0.0198 (14)	
H8	1.1509	0.2398	1.0921	0.024*	
C9	0.9991 (8)	0.2638 (6)	1.0179 (3)	0.0171 (14)	
C10	1.0694 (7)	0.1553 (7)	0.9856 (3)	0.0184 (10)	
C11	1.0772 (7)	0.0274 (6)	0.9004 (3)	0.0169 (13)	
C12	1.2176 (8)	−0.0386 (6)	0.9159 (3)	0.0198 (14)	
H12	1.2724	−0.0179	0.9514	0.024*	
C13	1.2770 (8)	−0.1323 (7)	0.8809 (3)	0.0230 (15)	
H13	1.3710	−0.1732	0.8933	0.028*	
C14	1.2006 (8)	−0.1695 (7)	0.8265 (3)	0.0201 (14)	
C15	1.0599 (7)	−0.1020 (6)	0.8098 (3)	0.0167 (13)	
H15	1.0055	−0.1227	0.7742	0.020*	
C16	1.0010 (8)	−0.0056 (7)	0.8452 (3)	0.0190 (14)	
H16	0.9091	0.0388	0.8325	0.023*	
C17	1.4009 (8)	−0.3421 (7)	0.8108 (3)	0.0236 (15)	
H17A	1.3981	−0.4306	0.7899	0.028*	
H17B	1.4031	−0.3645	0.8512	0.028*	
C18	1.5448 (9)	−0.2657 (7)	0.8027 (4)	0.0333 (19)	
H18A	1.6310	−0.3233	0.8165	0.050*	
H18B	1.5498	−0.1786	0.8237	0.050*	
H18C	1.5457	−0.2462	0.7627	0.050*	
C19	1.1893 (9)	−0.2913 (7)	0.7336 (3)	0.0261 (16)	
H19A	1.2676	−0.3201	0.7107	0.031*	
H19B	1.1440	−0.2049	0.7171	0.031*	
C20	1.0669 (8)	−0.4057 (7)	0.7300 (3)	0.0262 (16)	
H20A	1.0250	−0.4197	0.6907	0.039*	
H20B	0.9874	−0.3770	0.7517	0.039*	
H20C	1.1112	−0.4923	0.7454	0.039*	
N1	0.8788 (6)	0.3370 (5)	0.9937 (2)	0.0136 (11)	
N2	1.0102 (6)	0.1260 (5)	0.9339 (2)	0.0158 (11)	
N3	1.2599 (6)	−0.2626 (5)	0.7915 (2)	0.0193 (12)	
Cl1	0.7217 (2)	0.37547 (19)	0.82157 (8)	0.0343 (5)	
Cl2	0.61076 (19)	0.05105 (18)	0.93193 (7)	0.0275 (4)	
Hg1	0.77787 (3)	0.23825 (3)	0.905699 (10)	0.01999 (11)	
H10C	1.155 (5)	0.115 (8)	1.004 (3)	0.04 (2)*	

### Atomic displacement parameters (Å^2^)

**Table:**

	*U*^11^	*U*^22^	*U*^33^	*U*^12^	*U*^13^	*U*^23^
C1	0.013 (2)	0.019 (2)	0.026 (3)	−0.0041 (18)	0.014 (2)	0.0005 (19)
C2	0.020 (4)	0.024 (4)	0.022 (4)	−0.006 (3)	−0.002 (3)	−0.001 (3)
C3	0.022 (4)	0.016 (3)	0.039 (4)	−0.007 (3)	0.008 (3)	−0.007 (3)
C4	0.019 (4)	0.024 (4)	0.025 (4)	−0.006 (3)	0.010 (3)	−0.002 (3)
C5	0.034 (4)	0.025 (4)	0.020 (4)	−0.011 (3)	0.000 (3)	−0.004 (3)
C6	0.028 (4)	0.013 (3)	0.025 (4)	−0.006 (3)	0.003 (3)	0.000 (3)
C7	0.021 (4)	0.018 (3)	0.024 (4)	−0.011 (3)	0.005 (3)	0.001 (3)
C8	0.017 (3)	0.028 (4)	0.012 (3)	−0.003 (3)	−0.006 (3)	0.002 (3)
C9	0.019 (4)	0.019 (3)	0.013 (3)	−0.010 (3)	0.001 (3)	0.001 (2)
C10	0.013 (2)	0.019 (2)	0.026 (3)	−0.0041 (18)	0.014 (2)	0.0005 (19)
C11	0.021 (4)	0.007 (3)	0.021 (3)	0.001 (3)	−0.002 (3)	−0.001 (2)
C12	0.024 (4)	0.013 (3)	0.022 (3)	−0.007 (3)	0.001 (3)	0.000 (3)
C13	0.018 (4)	0.014 (3)	0.036 (4)	0.003 (3)	−0.002 (3)	0.004 (3)
C14	0.021 (4)	0.022 (4)	0.017 (3)	−0.005 (3)	0.001 (3)	−0.001 (3)
C15	0.019 (3)	0.013 (3)	0.016 (3)	−0.003 (3)	−0.005 (3)	0.002 (2)
C16	0.020 (3)	0.014 (3)	0.022 (4)	0.005 (3)	−0.001 (3)	0.005 (3)
C17	0.022 (4)	0.015 (3)	0.033 (4)	0.004 (3)	0.003 (3)	−0.003 (3)
C18	0.022 (4)	0.024 (4)	0.054 (5)	0.006 (3)	0.006 (4)	0.008 (3)
C19	0.038 (4)	0.015 (3)	0.027 (4)	0.003 (3)	0.013 (3)	−0.001 (3)
C20	0.031 (4)	0.028 (4)	0.016 (3)	−0.004 (3)	−0.008 (3)	−0.004 (3)
N1	0.014 (3)	0.013 (3)	0.014 (3)	−0.008 (2)	0.002 (2)	−0.001 (2)
N2	0.013 (3)	0.016 (3)	0.018 (3)	0.002 (2)	0.002 (2)	0.002 (2)
N3	0.018 (3)	0.017 (3)	0.025 (3)	0.002 (2)	0.010 (2)	0.000 (2)
Cl1	0.0427 (12)	0.0243 (9)	0.0308 (10)	−0.0061 (8)	−0.0144 (8)	0.0075 (7)
Cl2	0.0202 (9)	0.0320 (9)	0.0285 (9)	−0.0071 (7)	−0.0035 (7)	0.0050 (7)
Hg1	0.01749 (17)	0.02169 (16)	0.02002 (16)	0.00000 (10)	−0.00049 (11)	−0.00019 (10)

### Geometric parameters (Å, º)

**Table:**

Hg1—Cl1	2.3654 (19)	C14—C15	1.410 (9)
Hg1—Cl2	2.4394 (18)	C15—C16	1.383 (9)
Hg1—N1	2.340 (5)	C17—C18	1.499 (10)
Hg1—N2	2.334 (5)	C19—C20	1.527 (10)
N1—C1	1.363 (8)	C2—H2	0.9300
N1—C9	1.335 (8)	C3—H3	0.9300
N2—C10	1.290 (8)	C4—H4	0.9300
N2—C11	1.402 (8)	C5—H5	0.9300
N3—C14	1.358 (8)	C7—H7	0.9300
N3—C17	1.478 (9)	C8—H8	0.9300
N3—C19	1.448 (9)	C10—H10C	0.91 (6)
C1—C2	1.419 (10)	C12—H12	0.9300
C1—C6	1.418 (10)	C13—H13	0.9300
C2—C3	1.372 (10)	C15—H15	0.9300
C3—C4	1.392 (10)	C16—H16	0.9300
C4—C5	1.368 (10)	C17—H17A	0.9700
C5—C6	1.410 (10)	C17—H17B	0.9700
C6—C7	1.397 (10)	C18—H18A	0.9600
C7—C8	1.373 (9)	C18—H18B	0.9600
C8—C9	1.440 (10)	C18—H18C	0.9600
C9—C10	1.464 (9)	C19—H19A	0.9700
C11—C12	1.397 (9)	C19—H19B	0.9700
C11—C16	1.418 (10)	C20—H20A	0.9600
C12—C13	1.362 (9)	C20—H20B	0.9600
C13—C14	1.412 (10)	C20—H20C	0.9600
			
Cl1—Hg1—Cl2	122.85 (6)	C3—C2—H2	120.00
Cl1—Hg1—N1	122.31 (12)	C2—C3—H3	120.00
Cl1—Hg1—N2	124.75 (13)	C4—C3—H3	120.00
Cl2—Hg1—N1	103.99 (13)	C3—C4—H4	119.00
Cl2—Hg1—N2	97.92 (13)	C5—C4—H4	120.00
N1—Hg1—N2	73.11 (17)	C4—C5—H5	120.00
Hg1—N1—C1	128.9 (4)	C6—C5—H5	120.00
Hg1—N1—C9	111.2 (4)	C6—C7—H7	120.00
C1—N1—C9	119.4 (5)	C8—C7—H7	120.00
Hg1—N2—C10	113.9 (4)	C7—C8—H8	121.00
Hg1—N2—C11	124.4 (4)	C9—C8—H8	120.00
C10—N2—C11	121.5 (5)	N2—C10—H10C	125 (4)
C14—N3—C17	121.5 (5)	C9—C10—H10C	116 (5)
C14—N3—C19	122.6 (6)	C11—C12—H12	119.00
C17—N3—C19	115.9 (5)	C13—C12—H12	119.00
N1—C1—C2	118.9 (6)	C12—C13—H13	119.00
N1—C1—C6	121.9 (6)	C14—C13—H13	119.00
C2—C1—C6	119.1 (6)	C14—C15—H15	119.00
C1—C2—C3	120.4 (6)	C16—C15—H15	119.00
C2—C3—C4	120.1 (6)	C11—C16—H16	119.00
C3—C4—C5	121.0 (6)	C15—C16—H16	119.00
C4—C5—C6	120.7 (6)	N3—C17—H17A	109.00
C1—C6—C5	118.6 (6)	N3—C17—H17B	109.00
C1—C6—C7	118.3 (6)	C18—C17—H17A	109.00
C5—C6—C7	123.0 (6)	C18—C17—H17B	109.00
C6—C7—C8	119.9 (6)	H17A—C17—H17B	108.00
C7—C8—C9	119.1 (6)	C17—C18—H18A	110.00
N1—C9—C8	121.4 (6)	C17—C18—H18B	109.00
N1—C9—C10	120.9 (6)	C17—C18—H18C	109.00
C8—C9—C10	117.7 (6)	H18A—C18—H18B	109.00
N2—C10—C9	119.5 (6)	H18A—C18—H18C	109.00
N2—C11—C12	125.4 (6)	H18B—C18—H18C	109.00
N2—C11—C16	118.4 (6)	N3—C19—H19A	109.00
C12—C11—C16	116.2 (6)	N3—C19—H19B	109.00
C11—C12—C13	122.3 (6)	C20—C19—H19A	109.00
C12—C13—C14	122.2 (7)	C20—C19—H19B	109.00
N3—C14—C13	122.3 (6)	H19A—C19—H19B	108.00
N3—C14—C15	121.5 (6)	C19—C20—H20A	110.00
C13—C14—C15	116.2 (6)	C19—C20—H20B	109.00
C14—C15—C16	121.4 (6)	C19—C20—H20C	109.00
C11—C16—C15	121.7 (6)	H20A—C20—H20B	109.00
N3—C17—C18	114.4 (6)	H20A—C20—H20C	109.00
N3—C19—C20	113.7 (6)	H20B—C20—H20C	109.00
C1—C2—H2	120.00		
			
C6—C1—C2—C3	−0 (1)	N2—C11—C16—C15	−179.8 (6)
N1—C1—C2—C3	−179.0 (6)	C12—C11—N2—C10	−6 (1)
C2—C1—C6—C5	2 (1)	C12—C11—N2—Hg1	178.5 (5)
C2—C1—C6—C7	−179.1 (6)	C16—C11—N2—C10	176.3 (6)
N1—C1—C6—C5	−179.6 (6)	C16—C11—N2—Hg1	1.1 (8)
N1—C1—C6—C7	−0 (1)	C11—C12—C13—C14	−0 (1)
C2—C1—N1—C9	177.2 (6)	C12—C13—C14—C15	1 (1)
C2—C1—N1—Hg1	−12.2 (9)	C12—C13—C14—N3	179.8 (6)
C6—C1—N1—C9	−1.7 (9)	C13—C14—C15—C16	−0 (1)
C6—C1—N1—Hg1	169.0 (5)	N3—C14—C15—C16	−179.0 (6)
C1—C2—C3—C4	−1 (1)	C13—C14—N3—C17	7 (1)
C2—C3—C4—C5	1 (1)	C13—C14—N3—C19	−174.1 (6)
C3—C4—C5—C6	1 (1)	C15—C14—N3—C17	−174.9 (6)
C4—C5—C6—C1	−2 (1)	C15—C14—N3—C19	5 (1)
C4—C5—C6—C7	178.9 (7)	C14—C15—C16—C11	−2 (1)
C1—C6—C7—C8	2 (1)	C18—C17—N3—C14	−86.3 (8)
C5—C6—C7—C8	−178.5 (7)	C18—C17—N3—C19	94.3 (7)
C6—C7—C8—C9	−2 (1)	C20—C19—N3—C14	−86.4 (8)
C7—C8—C9—C10	178.8 (6)	C20—C19—N3—C17	93.1 (7)
C7—C8—C9—N1	0 (1)	C1—N1—Hg1—N2	178.5 (6)
C8—C9—C10—N2	178.7 (6)	C1—N1—Hg1—Cl1	57.6 (5)
N1—C9—C10—N2	−3 (1)	C1—N1—Hg1—Cl2	−87.3 (5)
C8—C9—N1—C1	1.5 (9)	C9—N1—Hg1—N2	−10.3 (4)
C8—C9—N1—Hg1	−170.7 (5)	C9—N1—Hg1—Cl1	−131.1 (4)
C10—C9—N1—C1	−176.8 (6)	C9—N1—Hg1—Cl2	83.9 (4)
C10—C9—N1—Hg1	11.1 (7)	C10—N2—Hg1—N1	9.2 (4)
C9—C10—N2—C11	177.2 (6)	C10—N2—Hg1—Cl1	127.2 (4)
C9—C10—N2—Hg1	−7.1 (8)	C10—N2—Hg1—Cl2	−93.0 (4)
C16—C11—C12—C13	−2 (1)	C11—N2—Hg1—N1	−175.2 (5)
N2—C11—C12—C13	−179.2 (6)	C11—N2—Hg1—Cl1	−57.2 (5)
C12—C11—C16—C15	2 (1)	C11—N2—Hg1—Cl2	82.6 (5)

### Hydrogen-bond geometry (Å, º)

**Table:**

*D*—H···*A*	*D*—H	H···*A*	*D*···*A*	*D*—H···*A*
C12—H12···Cl2^i^	0.93	2.82	3.703 (7)	159
C15—H15···Cl1^ii^	0.93	2.82	3.715 (7)	162

Symmetry codes: (i) −*x*+2, −*y*, −*z*+2; (ii) −*x*+3/2, *y*−1/2, −*z*+3/2.
